# Small non-coding RNA expression in mouse nephrogenic mesenchymal progenitors

**DOI:** 10.1038/sdata.2018.218

**Published:** 2018-11-13

**Authors:** Yu Leng Phua, Andrew Clugston, Kevin Hong Chen, Dennis Kostka, Jacqueline Ho

**Affiliations:** 1 Rangos Research Center, Children’s Hospital of Pittsburgh of UPMC, Pittsburgh, PA, USA; 2 Department of Pediatrics, Division of Nephrology, University of Pittsburgh School of Medicine, PA, USA; 3 Department of Developmental Biology, University of Pittsburgh School of Medicine, Pittsburgh, PA, USA; 4 Department of Computational & Systems Biology and Pittsburgh Center for Evolutionary Biology and Medicine, University of Pittsburgh School of Medicine, Pittsburgh, PA, USA

**Keywords:** Organogenesis, Paediatric research

## Abstract

MicroRNAs (miRNAs) are small non-coding RNAs that are essential for the regulation of gene expression and play critical roles in human health and disease. Here we present comprehensive miRNA profiling data for mouse nephrogenic mesenchymal progenitors, a population of cells enriched for nephron progenitors that give rise to most cell-types of the nephron, the functional unit of the kidney. We describe a miRNA expression in nephrogenic mesenchymal progenitors, with 162 miRNAs differentially expressed in progenitors when compared to whole kidney. We also annotated 49 novel miRNAs in the developing kidney and experimentally validated 4 of them. Our data are available as a public resource, so that it can be integrated into future studies and analyzed in the context of other functional and epigenomic data in kidney development. Specifically, it will be useful in the effort to shed light on molecular mechanisms underlying processes essential for normal kidney development, like nephron progenitor specification, self-renewal and differentiation.

## Background & Summary

MicroRNAs (miRNAs) are small, endogenously synthesized non-coding RNAs (~22 nucleotides long) that are critical in a wide variety of biological processes, where they primarily act to fine-tune gene expression at the post-transcriptional and translational level^1^. Biosynthesis of miRNAs begins in the nucleus, where a primary miRNA (pri-miRNA) transcript is first transcribed by RNA polymerase II^1,2^. Subsequently, the endoribonuclease enzyme Drosha-Dgcr8 complex processes pri-miRNAs into individual hairpin-shaped stem loop precursor miRNAs (pre-miRNAs), which are then exported into the cytoplasm via Exportin5^3–5^. In the final steps of miRNA biogenesis, Dicer processes pre-miRNA into mature miRNA, with the miRNA loaded into the RNA-induced silencing complex (RISC)^6–8^. mRNA 3’UTR target recognition by miRNA is primarily dependent on the miRNA seed sequence located within nucleotide position 2–8 of the miRNA, and complementary Watson-Crick base pairing between the miRNA and mRNA allows the RISC complex to catalyse the process of mRNA degradation and/or translational inhibition^9,10^.

Nephron progenitors are multipotent cells that undergo a mesenchymal to epithelial transition to subsequently differentiate into glomerular podocytes, proximal tubules, loops of Henle and distal tubule in the developing kidney^11^. They also self-renew throughout kidney development, and the number of nephron progenitors is one of the primary determinants of nephron endowment at birth^12^. Since no new nephrons are formed postnatally in humans, low nephron number increases an individual’s risk to develop chronic kidney disease in adulthood^13^. While the role of many protein-coding genes in nephron progenitor self-renewal, maintenance and differentiation is well established, the precise role of small non-coding RNAs remains ill-defined. There are several lines of evidence that point to the importance of miRNAs in kidney development and disease. Mutations in *DROSHA* and *DICER1* have been identified and implicated in several human kidney disorders, including paediatric Wilms Tumour and multilocular cystic renal tumours^14^. Conditional ablation of Dicer-dependent miRNAs in mouse nephron progenitor or ureteric bud lineages during renal development resulted in severe renal hypodysplasia (small kidneys) and collecting duct cyst formation, due to aberrant progenitor apoptosis and attenuated cilium length respectively^15,16^. Also, conditional ablation of Dicer or Drosha in glomerular podocytes and renal stroma results in early postnatal death and a wide variety of renal anomalies including podocyte foot process effacement, marked proteinuria, and glomerular aneurysms^17–21^.

**Figure 1:**
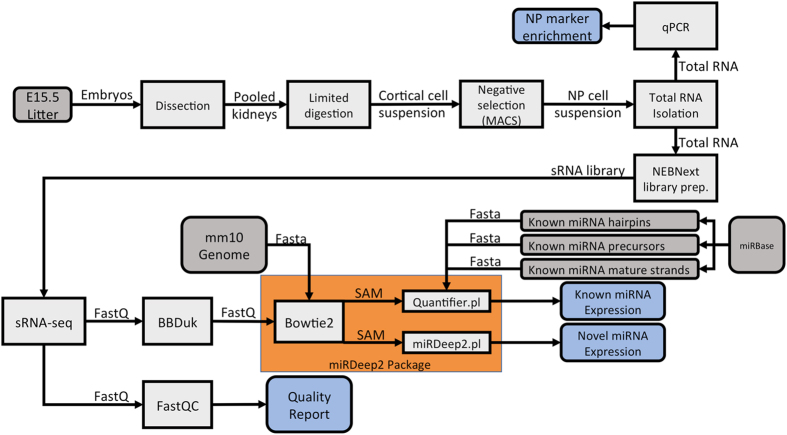
Schematic pipeline illustrating the workflow of nephron progenitor isolation and bioinformatics analysis of the small RNA-Seq dataset. For nephron progenitor isolation, embryonic day 15.5 (E15.5) CD1 mouse kidneys were subjected to limited digestion, followed by negative cell selection through Magnetic Activated Cell Sorting (MACS). Total RNA from the isolate was extracted and subjected to qPCR analysis for enrichment of nephron progenitor markers. Following verification of nephron progenitor enrichment, total RNA was used as an input for the NEBNextMultiplex Small RNA Library Prep to generate libraries for the sRNA-Seq. The sRNA-Seq dataset was analysed in accordance with the pipeline, with the fastq files first analysed by FastQC to determine the quality of the sequencing reads, followed by adaptor removal using the *BBDuk* package, and finally aligned, quantified and annotated to the *mus musculus* mm10 genome using the miRDeep2 package.

However, more precise characterisation of the role of small RNAs in nephron progenitors during kidney development has been hampered by the lack of comprehensive miRNA expression datasets in this context. Given the biomedical relevance of this system, we have performed high throughput small RNA Sequencing (sRNA-Seq) in three biological replicates of embryonic day 15.5 (E15.5) nephrogenic mesenchymal cells enriched for nephron progenitors and whole kidney (Fig. 1). Using an adjusted p-value cutoff of 0.05, we identified a total of 162 miRNAs (5p and 3p strand inclusive) out of 792 detectable miRNAs to be differentially expressed in this population when compared to whole kidney. Among the top differentially expressed miRNAs are members of the epithelial-specific *miR-200* family, consisting of *miRs-200a, 200b, 200c, 141* and *429*^22^. Levels of *miR-200* family miRNAs^22^ were significantly lower in nephron progenitors, as might be predicted given that nephron progenitors undergo a mesenchymal to epithelial transition upon differentiation. Furthermore, we uncovered 49 novel miRNA species expressed in the developing kidney. Of these miRNAs, 4 were validated via quantitative real-time PCR (qPCR), with 3 via section *in situ* hybridization. In general, the data resource will be useful for researchers studying miRNA related biology in kidney/nephron development.

## Methods

### Nephron progenitor isolation and total RNA preparation

Nephrogenic mesenchymal cells enriched for nephron progenitors and whole kidney samples were isolated from 3 litters of E15.5 CD1 mouse embryos (Charles River Laboratories) in accordance to a published protocol using a negative selection approach^23^. Briefly, intact embryonic kidneys were subjected to limited digestion, followed by incubation with a cocktail of monoclonal biotinylated antibodies (eBioscience; CD140a #13-1401-82, CD105 #13-1051-82, Epcam #13-5791-82 and Ter119 #13-5921-82), and magnetic activated cell sorted using Dynabeads MyOne Streptavidin C1 magnetic beads (Thermo; #65001) to deplete unwanted cell types (Fig. 1). To minimise undesired gene expression changes, total RNA from nephron progenitors were immediately processed in QIAzol Lysis Reagent (Qiagen; #79306) and purified using a miRNeasy Micro Kit (Qiagen; #217084). For whole kidney samples, remnant kidneys left from the limited digestion step were homogenised in QIAzol Lysis Reagent, clarified by centrifugation at 13,000 rpm for 5 min, and subsequently purified using a miRNeasy Mini Kit (Qiagen; #217004). Purified total RNA samples were stored at -80 °C until further processing, and freeze thawing of samples was limited to no more than 2 cycles. Quantitative PCR (qPCR) was carried out using standard SYBR Green detection on a BioRad CFX96 Real Time PCR Instrument to determine enrichment of nephron progenitors from the isolation. Primers used include *Six2, Cited1* (nephron progenitors), *Pdgfrβ* (renal stroma), *VE-Cad* (endothelial)*, Calb, Epcam* (epithelial tubules) and *Synpo* (podocytes) (Table 1). Statistical analysis was performed using an unpaired Student’s t-test, and genes with a p-value of <0.05 were considered statistically significant.

To account for biological heterogeneity, a total of 3 biological replicates were collected from 3 litters of wildtype CD1 embryos. All experimental procedures were performed in accordance with the University of Pittsburgh Institutional Animal Care and Use Committee guidelines, which adheres to the NIH Guide for the Care and Use of Laboratory Animals. sRNA-Seq experimental workflow and quality control standards were carried out in accordance with ENCODE guidelines (https://www.encodeproject.org/rna-seq/small-rnas/).

### Small RNA library preparation and sequencing

Because miRNAs can be stably bound to mRNAs^24^, total RNA instead of size-selected purified small RNA was used as the initial input for cDNA library synthesis. The NEBNextMultiplex Small RNA Library Prep Set for Illumina (NEB; #E7300S) was used to synthesise barcoded cDNA libraries from 100 ng total RNA in accordance with the manufacturer’s protocol and size selection performed using the Agencourt AMPure XP beads (Beckman Coulter; #A63881). The purified cDNA libraries were then pooled, normalised and multiplex sequenced at 1 X 50 bp with the use of a NextSeq 500/550 Mid Output v2 Kit (150 cycles) (Illumina; FC-404-2001) on an Illumina NextSeq 550 System at the Rangos Research Center, yielding approximately 18 million reads per sample.

### Small RNA-Seq data analysis and quality control

Adapter trimming was performed using the BBmap software (https://sourceforge.net/projects/bbmap/) and reads were aligned to the mouse genome (mm10) using Bowtie2^25^. miRNA specific analysis was performed with miRDeep2 software^26^. Expression of known miRNAs (obtained from miRBase v21^27^) was quantified, and miRNAs with more than 10 counts across all sample conditions were subsequently analysed using DESeq2^28^ to identify differential expression between nephron progenitor and whole kidney samples. miRNAs with an Benjamini-Hochberg adjusted p-value of 5% or less are reported. Novel miRNAs were annotated with the standard miRDeep2 parameters, and read alignment data was visualized with the Integrative Genomics Viewer software available from the Broad Institute^29–32^.

### Data Records

The sRNA-Seq fastq files were deposited at NCBI Sequence Read Archive (Data Citation 1). This accession contains all 6 fastq files from the sRNA-Seq. The raw fastq files were subsequently processed and deposited into NCBI Gene Expression Omnibus (Data Citation 2).

### miRNA Quantitative PCR and Locked Nucleic Acid in situ hybridization validation

For the validation of differentially expressed miRNAs, cDNA synthesis was carried out with the use of TaqMan Advanced miRNA cDNA Synthesis Kit (Thermo; #A28007) in accordance to the manufacturer’s protocol using equivalent amounts of total RNA. qPCR was carried out with the use of TaqMan Advanced miRNA Assay probes (Thermo; #A25576; 477952_miR, 477970_miR, 477885_miR, 478008_miR) and TaqMan Universal Master Mix II, no UNG (Thermo; #4440040) on a BioRad CFX96 Real Time PCR Instrument using FAM detection.

For the validation of novel miRNAs, cDNA synthesis from total RNA was performed with the use of NCode VILO miRNA cDNA Synthesis Kit (Thermo; #A11193050) in accordance to the manufacturer’s protocol using equivalent amounts of total RNA, and qPCR was performed using standard SYBR Green detection on a BioRad CFX96 Real Time PCR Instrument. The universal reverse primer is supplied with the NCode VILO miRNA cDNA Synthesis Kit, and the forward primer sequence is listed in Table 2. Locked Nucleic Acid miRNA *in situ* hybridisation on E15.5 embryonic kidney cryosections was performed as previously described with the use of custom-designed LNA detection probes^19^ (Table 3) (Exiqon).

### Code Availability

Software and settings used in the processing of this data are listed as follows: 1) BBDuk: from the BBMap version 35.59 package, adapter references for Illumina indices and the Nextera smRNA library prep set, quality trimming right to left (qtrim=30), kmer trimming right to left (ktrim=r) then left to right (ktrim=l), minimum kmers of 11 (mink=11), hamming distance of 1 bp (hdist=1), minimum final read length of 18 bp (minlen=18). 2) Bowtie 2: version 2.2.3, parameters of –threads 8, -q, -U, -S, --sensitive-local. 3) miRDeep2 version 2.0.0.8, using the bwa_sam_converter.pl script to convert Bowtie2’s SAM output formats, and the miRDeep2.pl script using default parameters for the mouse species (-t Mouse) and miRbase v18 identifiers (-P). Reference data included a pre-built Bowtie2 index for the mm10 genome and miRNA mature and hairpin sequences available in Illumina’s mm10 iGenomes package. A FASTA with miRNA “other” sequences were provided from miRbase version 21. 4) R: version 3.4.3, data analysis was performed using the R programming language, including packages mirbase.db, DESeq2, and GenomicRanges.

## Technical Validation

### Cellular enrichment and total RNA quality control

**Figure 2:**
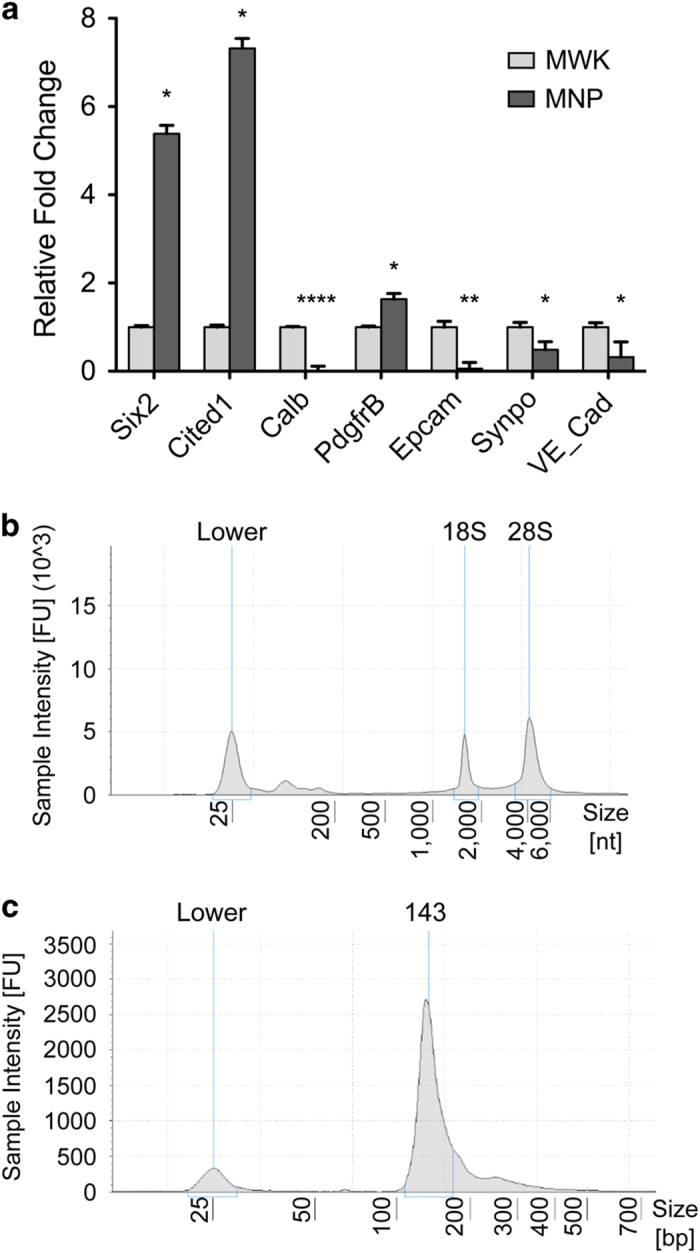
Quality control of the input samples demonstrated an enrichment of nephron progenitors in the cellular isolate with good quality RNA, and successful cDNA library synthesis. (**a**) qPCR analysis showed that the cells in the isolate are enriched for nephron progenitors (*Six2, Cited1*) with minimal cellular contamination from ureteric bud (*Calb*), podocytes (*Synpo*), endothelial (*VE-Cad*) and epithelial cells (*Epcam*), but minor contamination from the renal stroma lineage (*Pdgfrβ*). (**b**) TapeStation analysis of the total RNA showed good quality RNA traces with distinct 18S and 28S rRNA peaks with retention of small RNAs as evident by the broad peak to the right of the lower molecular marker. (**c**) TapeStation analysis showed that the purified cDNA libraries exhibited a distinct peak at ~140–150 nucleotides (adaptor ligated small RNA products), indicating that both the cDNA library construction and cleanup are successful. N=3, ^∗^p-value<0.05, ^∗∗^p-value<0.01, ^∗∗^^∗∗^p-value<0.0001.

Our overall goal was to quantify small RNA expression in mouse nephron progenitors and whole kidney. We used a previously published protocol^23^ to obtain an enriched fraction of nephron progenitor cells from whole embryonic kidney samples. Confirming that this approach was successful, qPCR analysis of the nephron progenitor-enriched isolate showed high enrichment of nephron progenitor transcripts (*Six2*, *Cited1*) in comparison to whole kidney samples, with evidence of minimal cellular contamination from other key renal cell lineages including the endothelium (*VE-Cad*), epithelial tubules (*Calb, Epcam*) and podocytes (*Synpo*) (Fig. 2a). An inherent limitation of this protocol^23^ is minor contamination from the renal stroma (*Pdgfrβ*), which was indeed detected in our isolate. Although the Six2-TGC transgenic mouse^33^ provides the opportunity for isolating a more pure fraction of nephron progenitor cells, these mice are known to have renal hypoplasia^34^, suggesting that their nephron progenitors may not be entirely normal. Taken together, our small RNA sequencing dataset represents a highly-enriched fraction of wild-type nephron progenitors, with minor contamination from renal stroma.

Following cellular isolation and total RNA extraction, the quality of the total RNA was assessed on an Agilent 4200 TapeStation System (Agilent; #G2991AA) using a High Sensitivity RNA ScreenTape (Agilent; #5067-5579) to determine the RNA Integrity Number (RIN) score, which is generated based on the electrophoretic profile of 18S and 28S ribosomal RNA (rRNA)^35^ (Fig. 2b). All RNA samples used for the sRNA-Seq had a RIN score of above 9 (Table 4) and were therefore considered to be good quality intact RNA with minimal degradation. Retention of small RNA in the total RNA isolation was also evident by a distinct broad peak to the right of the lower molecular marker (Fig. 2b). To ensure equivalent amounts of total RNA input for the cDNA library construction, the Qubit RNA BR Assay Kit (Thermo; #Q10210) was used for RNA quantitation.

### Quality control of the cDNA libraries

cDNA libraries were assayed on an Agilent 4200 TapeStation System (Agilent; #G2991AA) using a High Sensitivity D1000 ScreenTape (Agilent; #5067-5584) and were verified to exhibit a distinct peak at ~140-150 nucleotides that corresponds to adapter-ligated constructs derived from small RNAs (Fig. 2c). Moreover, TapeStation analysis verified that the final cDNA libraries for sequencing were depleted of large molecular weight products that one would expect be indicative of larger RNA molecules like messenger or ribosomal RNA.

### Quality control of sRNA-Seq data

**Figure 3:**
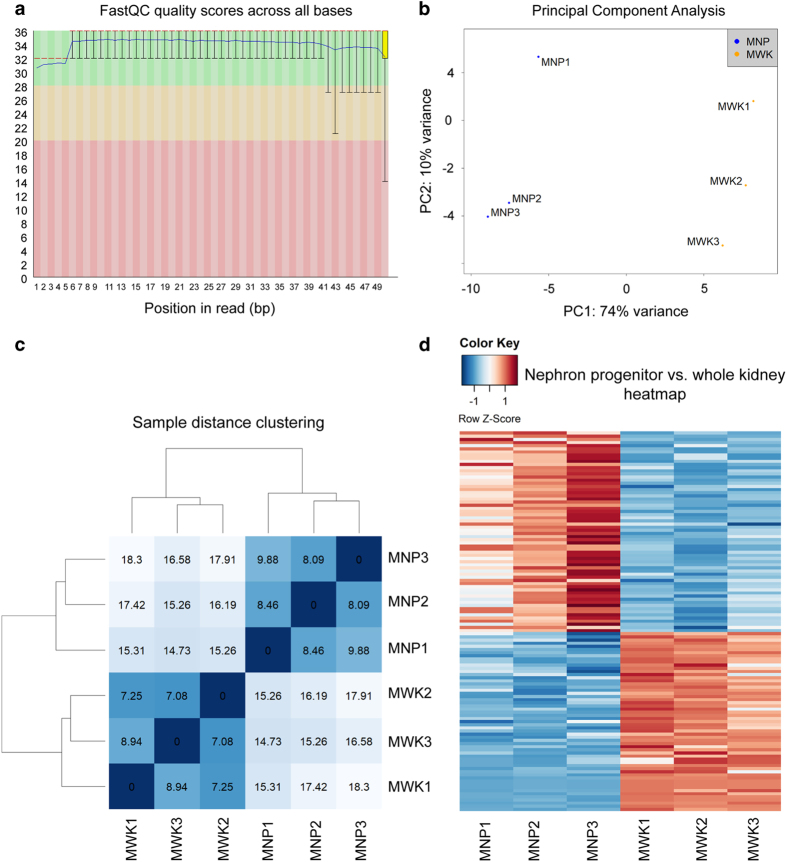
Quality control of the small RNA-Seq dataset showed good sequencing quality reads and congruence of the biological samples. (**a**) FastQC analysis of a sequenced library (MNP1) showing that each sequenced base had a mean value score of >30, indicating good quality sequencing. (**b**) Principal Component Analysis of the sRNA-Seq dataset showed clear separation of the nephron progenitor (MNP) and whole kidney (MWK) samples. (**c**) Hierarchical clustering correctly grouped samples based on their tissue of origin. (**d**) Heatmap representation of differentially expressed miRNAs in nephron progenitors vs. whole kidney samples with a false discovery rate of 0.05.

Purified cDNA libraries for all samples were normalised and multiplex sequenced using a single flow cell on the Illumina NextSeq550 System to minimise technical variability from the sequencing. Following sequencing, reads for each sample was evaluated using the FastQC software (https://www.bioinformatics.babraham.ac.uk/projects/fastqc/) and all sequenced libraries had an average quality score of above 30, indicating high quality base of the sequenced product (Fig. 3a). Percentage of aligned reads was consistently above 70%, with 13 million reads mapped per sample on average. For miRDeep2 analysis, aligned reads shorter than 18 bp were discarded in keeping with the software’s requirements^26^. Principal component analysis (PCA) revealed a clear distinction between and separation of the nephron progenitor (MNP) and whole kidney (MWK) samples (Fig. 3b), as did hierarchical clustering of samples based on their miRNA abundances (Fig. 3c). PCA and hierarchical clustering analysis both indicate that samples enriched for nephron progenitors are transcriptionally distinct from whole kidney samples.

**Figure 4:**
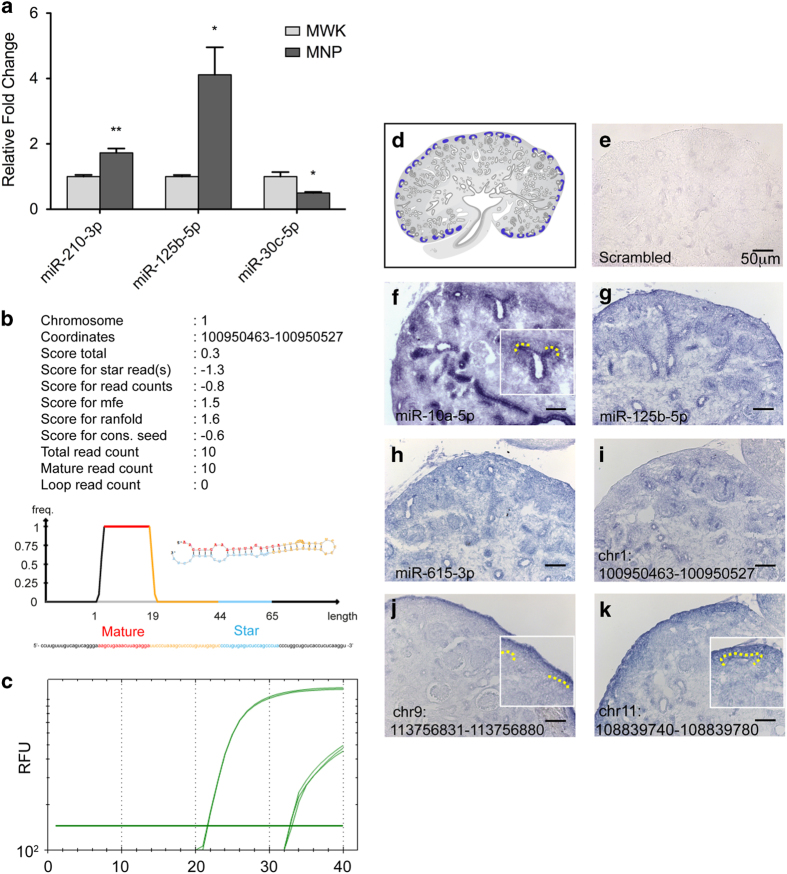
Small RNA-Seq and miRDeep2 analysis revealed novel unannotated miRNAs expressed in the developing kidney. (**a**) qPCR analysis validated the differential expression profile of *miRs-210, 125b* and *30c* in nephron progenitor when compared to whole kidney. N=3, ^∗^p-value<0.05, ^∗∗^p-value<0.01. (**b**) miRDeep2 output for chr1_100950463-10095052, a novel miRNA discovered in our sRNA-Seq data; high frequency (freq) of reads mapped to the mature region of the predicted pre-miRNA structure. (**c**) qPCR validation of chr1_100950463-100950527 showed a clear distinction between and enrichment of mature (low C_T_) over star strand (High C_T_) transcripts. RFU: Relative Fluorescence Unit. (**d**) For spatial orientation purposes, schematic representative image of nephron progenitors is highlighted in blue. (**e**-**k**) Locked Nucleic Acid section *in situ* hybridisation was used to validate the spatial expression pattern of miRNAs in the developing kidney. *miRs-125b, 615* and chr1_100950463-100950527 exhibited a ubiquitous expression pattern in the developing kidney. *miR-10a*, chr9_113756831-113756880 and chr11_108839740-108839780 appear spatially enriched in the nephrogenic zone, comprising nephron progenitors, renal stroma and the ureteric bud. Schematic image in (**d**) was designed by Kylie Georgas (University of Queensland) and are publicly available for use from the GUDMAP database (http://www.gudmap.org/Schematics).

We identified a total of 162 miRNAs (5p and 3p strand inclusive) out of 792 detectable miRNAs to be statistically differentially expressed in nephrogenic mesenchymal cells enriched for nephron progenitors relative to whole kidney (Fig. 3d and Differentially expressed miRNAs in nephron progenitors & miRNAs expressed in kidney, Data Citation 3), and validated the differential profile of *miR-210, miR-125b* and *miR-30c* by qPCR (Fig. 4a). Among the top differentially expressed miRNAs are members of the epithelial-specific *miR-200* family, consisting of *miRs-200a, 200b, 200c, 141* and *429*^22^. Considering that nephron progenitors are mesenchymal in nature and that they undergo a mesenchymal to epithelial transition upon differentiation^33^, the under-expression of the *miR-200* cluster in nephron progenitors not only serves as an excellent internal validation of the phenotypic characteristics of these cells, but also supports the overall integrity of the miRNA profiling dataset.

We explored the use of alternative bioinformatics approaches and obtained comparable results with choices such as *Rsubread* for read mapping to the mm10 genome (parameters: maximum allowed for 3 nucleotides mismatch and 1 insertion-deletion)^36^, *featureCounts* which outputs number of reads assigned to genomic features^37^ and *limma-voom* for differential expression statistical analysis^38^.

### Locked Nucleic Acid section in situ hybridisation and qPCR validation of the small RNA-Seq data

The additional novelty of this dataset stems from its inherent ability to identify novel unannotated miRNA transcripts without *a priori* sequence knowledge. A major challenge in novel miRNA identification is that presumed novel miRNA transcripts are in fact byproducts resulting from degraded mRNA transcripts, and hence not necessarily representative as *bona fide* novel miRNAs. To overcome this, the miRDeep2 package^26^ was used because it includes a series of stringent core analysis modules including the RNAfold tool (http://rna.tbi.univie.ac.at/) and the miRDeep2 core algorithm. The RNAfold tool determines and predicts whether the novel miRNA sequence would exhibit a typical hairpin secondary structure, and the miRDeep2 core algorithm evaluates both the miRNA’s secondary structure and the expected read mapping signature of its hairpin precursor. The core algorithm also checks that the sequencing reads which span the predicted hairpin structure contains specific recognition sites for subsequent processing by Dicer into mature miRNAs. Novel miRNAs were considered high confidence when both mature and star strands were detected in at least 2 independent samples, in conjunction with sequencing reads spanning across the putative chromosome coordinates independently verified on IGV Viewer. In this manner, we identified a total of 49 novel miRNAs, exemplified by the 20 nucleotides long novel miRNA (GGA AAG CTG AAA CTT AGA GG) depicted with a characteristic hairpin precursor secondary structure located within chr1: 100950463-100950527 (Fig. 4b and Novel miRNAs expressed in kidney, Data Citation 3). These novel miRNAs will be submitted for names through miRBase in accordance to their criteria.

As a confirmation that the novel miRNAs were not an artefact from sRNA sequencing, qPCR was performed on 4 candidate novel miRNAs. The qPCR analysis depicted distinct detection of mature (low C_T_) over star strand (high C_T_) transcripts (Fig. 4c and Table 5), thereby validating the overall approach in novel miRNA discovery through sRNA-Seq and miRDeep2 identification. Using miRNA Locked Nucleic Acid *in situ* hybridization for spatial localisation of miRNA expression sites, both annotated (Fig. 4f–h) (*miRs-10a, 125b, 615*) and novel miRNAs (Fig. 4i–k) (chr1_100950463- 100950527, chr9_113756831-113756880, chr11_108839740-108839780) were found to be expressed in the developing mouse kidney. *miR-10a*, chr9_113756831-113756880 and chr11_108839740-108839780 exhibited a distinct expression pattern within the nephrogenic zone, encompassing nephron progenitors, renal stroma and the ureteric bud epithelium. Together, these assays validate both the sRNA-Seq dataset and the approach for novel miRNA discovery in the developing mouse kidney.

## Usage Notes

The sRNA-Seq data presented here represents a comprehensive resource for miRNA expression in developing nephrogenic mesenchymal cells enriched for nephron progenitors and whole kidney. This resource can be utilised to predict regulatory networks downstream of these expressed miRNAs. For example, this dataset can be integrated with other published RNA-Seq data containing transcriptomic information (mRNA and long non-coding RNA) from nephron progenitors and whole kidney for the analysis of miRNA-mRNA interactions using algorithms such as TargetScan^39^ or DIANA-Tools^40^. Such analysis would allow one to uncover potential candidate miRNAs and/or downstream target mRNAs for future studies. For example, our sRNA-Seq dataset supports the expression of the highly conserved *let-7* family members (*let-7a, 7b, 7c, 7d, 7e, 7f, 7g, 7h, 7i, 7j, 7k* and *miR-98*) in the developing kidney with *let-7c, 7d, 7e* being differentially enriched in nephron progenitors compared to whole kidney during development. The pathogenesis of Wilms tumour has been linked to overexpression of Lin28, a negative regulator of *let-7* microprocessing^41^. Wilms tumour is the most common paediatric kidney cancer and arises from a failure of embryonic kidney tissues to terminally differentiate. Thus, de-repression of *let-7* downstream targets including *Hmga2*^42,43^*, Ras*^44^ and *Myc*^45^ oncogenes may be implicated in the malignant transformation of nephron progenitors in Wilms Tumour^41^. This integrative analysis approach allows for future hypothesis testing studies to elucidate the biological function of key miRNAs in nephron progenitors.

**Figure 5:**
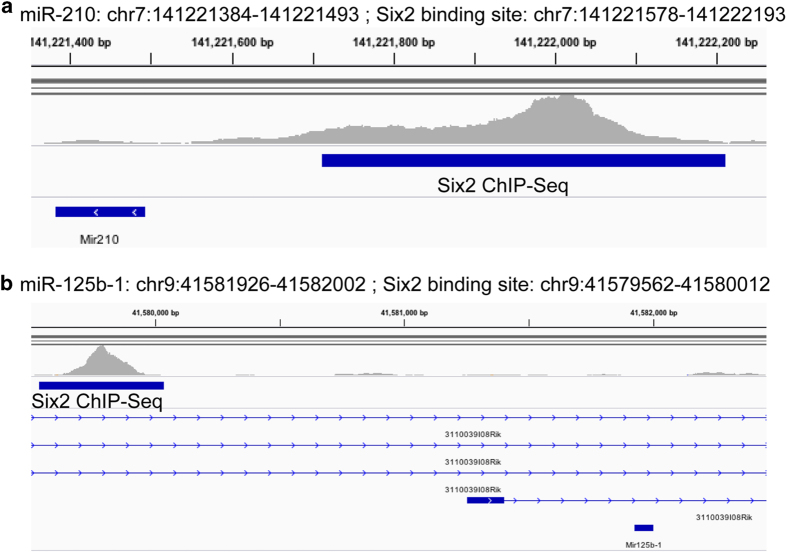
Integrative Genome Viewer visualisation of Six2 binding upstream of expressed miRNAs in nephron progenitors. By combining a publicly available Six2 ChIP-Seq dataset (SRA Study: SRP064623; GEO: GSE73867) with the current sRNA-Seq results, evidence for Six2 binding was observed in the promoter region of *miRs-210* and *125b*, miRNAs that are enriched in nephron progenitors.

Finally, the sRNA-Seq dataset can be complemented with published ChIP-Seq datasets to infer potential relationships between expressed miRNAs and *cis*-regulatory regions in nephron progenitors. The transcription factor Six2 is known to be critical in maintaining self-renewal and has been shown to regulate target genes that are indispensable for maintaining multipotency of these cells during nephrogenesis^46^. By analysing available Six2 ChIP-Seq data (SRA Study: SRP064623; GEO: GSE73867)^47^ with our current sRNA-Seq dataset, it is now possible to curate candidate miRNAs that are potentially regulated by Six2. Two examples are Six2 binding within the promoter regions of *miRs-210* and *125b*, miRNAs that are enriched in nephron progenitors based on this sRNA-Seq dataset (Fig. 5). This is an example of how to use this resource to identify putative Six2-regulated miRNAs that may play important roles in nephron progenitor maintenance and differentiation during kidney development.

## Additional information

**How to cite this article:** Phua, Y.L. *et al*. Small non-coding RNA expression in mouse nephrogenic mesenchymal progenitors. *Sci. Data*. 5:180218 doi: 10.1038/sdata.2018.218 (2018).

**Publisher’s note:** Springer Nature remains neutral with regard to jurisdictional claims in published maps and institutional affiliations.

## Supplementary Material



## Figures and Tables

**Table 1:** Primer sequences used for quantitative PCR.

**Gene**	**Forward (5′-3′)**	**Reverse (5′-3′)**
**Gapdh**	AGGTCGGTGTGAACGGATTTG	TGTAGACCATGTAGTTGAGGTCA
**Six2**	TCTGCTCGGTATCCTTTGGG	TTAAAAATCGGGGTGGTGGTG
**Cited1**	AACCTTGGAGTGAAGGATCGC	GTAGGAGAGCCTATTGGAGATGT
**Ve-Cad**	CACTGCTTTGGGAGCCTTC	GGGGCAGCGATTCATTTTTCT
**Pdgfrβ**	TTCCAGGAGTGATACCAGCTT	AGGGGGCGTGATGACTAGG
**Calb**	ATGATCAGGATGGCAACGGA	GTTCGGTACAGCTTCCCTCC
**Epcam**	GCGGCTCAGAGAGACTGTG	CCAAGCATTTAGACGCCAGTTT
**Synpo**	CCTGCCCGTAACTTCCGTG	GAGCGGCGGTAGGGAAAAG

**Table 2:** Primer sequences used for novel miRNA quantitative PCR.

**miRNA**	**Mature forward sequence (5′-3′)**	**Star forward sequence (5′-3′)**
**U6**	GTGCTCGCTTCGGCAGC	NA
**chr1_ 100950463- 100950527**	GCGGAAAGCTGAAACTTAGAGG	TGAGTCTCCAGCCCTACCCTAA
**chr8_ 66396466- 66396533**	GCGGTTCTTAGTTGGTGGAGC	GCGCTGATTTGACTGAGAATGT
**chr11_ 108839740- 108839780**	GCGTGAGAAGAACTTTGAAGAGC	GCGTCCTCAGAGTTAGTGCAGA
**chr13_ 23436971- 23437058**	GTCTAGGGGTATGATTCTCGCAA	GCGTTTTTATCGTGTTTCCTGT

**Table 3:** Exiqon miRCURY LNA detection probes used for miRNA section *in situ* hybridisation.

**LNA Probe**	**Product No.**	**Product sequence 5′-3′**
Scramble-miR	699003-300	Scrambled sequence
miR-125b-5p	611756-300	CACAAGTTAGGGTCTCAGGGA
miR-615-3p	615721-300	AAGAGGGAGACCCAGGCTC
miR-10a-5p	YD00612528	ACAAATTCGGATCTACAGGGTA
chr1_100950463- 100950527	Custom	CCTCTAAGTTTCAGCTTTCC
chr9_113756831- 113756880	Custom	GTCCTGTATTGTTATTTTT
chr11_108839740- 108839780	Custom	GCTCTTCAAAGTTCTTCTCA

**Table 4:** Sample IDs and RIN score of RNA samples used for small RNA-Sequencing.

**Sample**	**RIN**	**Fastq file name**
**MNP1**	10	MNP1.fastq
**MNP2**	10	MNP2.fastq
**MNP3**	10	MNP3.fastq
**MWK1**	9.9	MWK1.fastq
**MWK2**	9.8	MWK2.fastq
**MWK3**	10	MWK3.fastq

**Table 5:** Quantitative PCR profile of novel miRNA assayed.

**miRNA**	**Mature C_T_**	**Star C_T_**
**chr1_ 100950463- 100950527**	20.25	29.01
**chr8_ 66396466- 66396533**	7.04	37.65
**chr11_ 108839740- 108839780**	26.59	34.48
**chr13_ 23436971- 23437058**	20.60	30.65
